# Ceramides and sphingosine-1-phosphate mediate the distinct effects of M1/M2-macrophage infusion on liver recovery after hepatectomy

**DOI:** 10.1038/s41419-021-03616-9

**Published:** 2021-03-26

**Authors:** Hang Sun, Shibo Sun, Gang Chen, Haorong Xie, Sheng Yu, Xinxin Lin, Jianping Qian, Cungui Mao, Hongxian Peng, Hao Chen, Xuefang Chen, Yiyi Li, Cuiting Liu, Junmin Shi, Bili Zhu, Linghong Guo, Qingping Li, Pengxiang Huang, Yiran Wei, Xixin Huang, Meiqi Liu, Zhonglin Cui, Qifan Zhang, Jie Zhou, Chuanjiang Li, Kai Wang

**Affiliations:** 1 Division of Hepatobiliopancreatic Surgery, Department of General Surgery, Nanfang Hospital, Southern Medical University, Guangzhou, Guangdong China; 2 Department of Hepatopancreatobiliary Surgery, The Second Affiliated Hospital of Guangzhou University of Chinese Medicine, Guangzhou, Guangdong China; 3 The First Clinical College, Southern Medical University, Guangzhou, Guangdong China; 4 Department of Medicine and Cancer Center, The State University of New York at Stony Brook, Stony Brook, NY USA; 5 Department of Radiation Oncology, Nanfang Hospital, Southern Medical University, Guangzhou, Guangdong China; 6 Central Laboratory, Southern Medical University, Guangzhou, Guangdong China; 7 Huiqiao Department, Nanfang Hospital, Southern Medical University, Guangzhou, Guangdong China; 8 Department of Colorectal Surgery, Shanxi Cancer Hospital, Taiyuan, Shanxi China

**Keywords:** Cell growth, Metabolomics

## Abstract

Post-hepatectomy liver dysfunction is a life-threatening morbidity that lacks efficient therapy. Bioactive lipids involved in macrophage polarization crucially regulate tissue injury and regeneration. Herein, we investigate the key bioactive lipids that mediate the cytotherapeutic potential of polarized-macrophage for post-hepatectomy liver dysfunction. Untargeted lipidomics identified elevation of ceramide (CER) metabolites as signature lipid species relevant to M1/M2 polarization in mouse bone-marrow-derived-macrophages (BMDMs). M1 BMDMs expressed a CER-generation-metabolic pattern, leading to elevation of CER; M2 BMDMs expressed a CER-breakdown-metabolic pattern, resulting in upregulation of sphingosine-1-phosphate (S1P). After infusing M1- or M2-polarized BMDMs into the mouse liver after hepatectomy, we found that M1-BMDM infusion increased M1 polarization and CER accumulation, resulting in exaggeration of hepatocyte apoptosis and liver dysfunction. Conversely, M2-BMDM infusion enhanced M2 polarization and S1P generation, leading to alleviation of liver dysfunction with improved hepatocyte proliferation. Treatment of exogenous CER and S1P or inhibition CER and S1P synthesis by siRNA targeting relevant enzymes further revealed that CER induced apoptosis while S1P promoted proliferation in post-hepatectomy primary hepatocytes. In conclusion, CER and S1P are uncovered as critical lipid mediators for M1- and M2-polarized BMDMs to promote injury and regeneration in the liver after hepatectomy, respectively. Notably, the upregulation of hepatic S1P induced by M2-BMDM infusion may have therapeutic potential for post-hepatectomy liver dysfunction.

## Introduction

Hepatectomy is frequently performed in the management of a variety of benign and malignant liver diseases. Post-hepatectomy liver dysfunction caused by impaired liver recovery is still a life-threatening morbidity^1,2^. Due to lack of efficient therapy, the mortality of post-hepatectomy liver failure is reported at 50%^2^. Clinical and experimental hepatectomy elicits a centric role of macrophages in regulating injury and regeneration in the liver^3^. Currently, the functional plasticity of macrophages is characterized by tissue-damaging or repairing properties^4^. Classical (M1) polarization driven by lipopolysaccharide (LPS) and alternative polarization (M2) driven by interleukin-4 (IL-4) represent the extreme states associated with tissue injury and regeneration^5^. Elchaninov et al. reported that the increase of M2-like macrophages was likely correlated with liver regeneration after hepatectomy^6^. The study from Melgar-Lesmes et al. also demonstrated that the increased infiltration of M2 macrophages in the liver was linked to the enhancement of liver regeneration^7^. Notably, cytotherapy with polarized macrophages was found to alleviate certain hepatopathy by altering immune response in the liver, such as fibrosis^8^. These results altogether lead to a hypothesis that hepatoprotective macrophages which improve liver repair may have cytotherapeutic potential for post-hepatectomy liver dysfunction.

Emerging studies have demonstrated that bioactive lipids are critical in mediating the distinct functions of polarized macrophages in tissue damage and repair^9–12^. Giannakis et al. reported that dynamic changes in fatty acids support the phenotype transition of macrophages in muscle regeneration, providing substantial evidence for exploring lipid mediators in subtypes of macrophages which distinctly regulates tissue injury and regeneration^9^. Ceramides (CERs) and sphingosine-1-phosphate (S1P) in the sphingolipid metabolism are particularly highlighted to distinctly regulate hepatocyte injury and repair^13^. Accumulation of CER in hepatocytes has been reported to cause cell death and eventually lead to liver dysfunction, liver atrophy, and liver cancer^14–16^. In contrast to CER, S1P is a pro-survival lipid that is implicated in promoting tissue regeneration through enhancing cell proliferation and angiogenesis^17,18^. Lentsch and Hla’s studies demonstrated that extracellular S1P is important in promoting liver regeneration by enhancing angiogenesis and hepatocyte proliferation^19,20^. However, much remains unknown about the bioactive lipids in mediating the functions of polarized macrophages in post-hepatectomy liver recovery.

In this study, we investigated the role of bioactive lipids that mediated the effects of polarized-macrophages infusion on post-hepatectomy liver recovery. Our study uncovers CER and S1P as critical lipids that mediate the hepatotoxic and hepatoprotective effects of M1- and M2-macrophage infusion on liver recovery after hepatectomy, highlighting a cytotherapeutic capacity of M2 macrophage via generation of hepatoprotective S1P for post-hepatectomy liver dysfunction.

## Materials and methods

### Mouse model

C57BL/6 mice were bred and kept at the animal facility of Nanfang Hospital Southern Medical University on a 12 h/12 h light/dark cycle at 21 °C and 50–55% humidity under specific pathogen-free (SPF) conditions. Mouse 2/3 hepatectomy was performed as described by Mitchell and Willenbring^21^. The liver regeneration index was demonstrated using the ratio of the remnant liver weight and body weight. Liver tissues for immunohistochemistry were fixed in 4% paraformaldehyde (PFA) (Biosharp, Hefei, China) then embedded in paraffin or Tissue-Tek OCT compound (Sakura Finetek, Chuo-ku, Tokyo, Japan). Liver tissues for RNA isolation, protein extraction, or sphingolipid extraction were snap-frozen in liquid nitrogen then stored at −80 °C. Blood samples were allowed to clot at room temperature, and the serum was collected by centrifugution and then stored at −80 °C until analyzed. Serum total bilirubin (TBIL), alanine aminotransferase activity (ALT), and albumin (ALB) were determined using Bilirubin Assay Kit (Sigma, St. Louis, MO, USA), Alanine Transaminase Colorimetric Activity Assay Kit (Cayman Chemical, Ann Arbor, MI, USA), and Albumin Assay Kit (Sigma, St. Louis, MO, USA), respectively, following the manufacturer’s instructions. All animal experiments were carried out following the protocols approved by the Institutional Animal Care and Use Committee of Nanfang Hospital Southern Medical University (NFYY-2017-03).

### Bone-marrow-derived macrophages culture, small interfering RNA transfection, and BMDM infusion

Isolation, culture, and polarization of bone-marrow-derived macrophages (BMDMs) were performed according to the protocol published by Ying et al.^22^. BMDMs were incubated with phosphate buffer saline (PBS) (Gibco, Waltham, MA, USA) as vehicle control, 100 ng/ml LPS (Sigma, St. Louis, MO, USA), and 10 ng/ml IL-4 (Peprotech, Rocky Hill, NJ, USA) for 72 h to induce M1 and M2 polarization, respectively. For in vivo tracking after BMDM infusion, BMDM were transfected with green fluorescent protein (GFP) plasmid (Jikai, Shanghai, China) with Lipofectamine 2000 (Invitrogen, Waltham, MA, USA) before inducing polarization. For disturbing CER metabolism in polarized BMDMs, M1 and M2 BMDMs were transfected with small interfering RNA (siRNA) (Jikai, Shanghai, China) targeting Cers2 (5ʹ-CCUACACUGCACGAUGAUAUATT-3ʹ, 5ʹ-UAUAUCAUCGUGCAGUGUAGGTT-3ʹ) or Sphk1 (5ʹ-ACCUUCUUUCGCCUAGCAATT-3ʹ, 5ʹ-UUGCUAGGCGAAAGAAGGUTT-3ʹ) using X-treme GENE siRNA Transfection Reagent (Roche Diagnostics, Mannheim, Germany). Transfection was performed at 24 h before induction of polarization. Polarized BMDM infusion was performed as described^8,23^ with slight modification. Non-polarized (M0), M1, and M2 BMDMs were trypsinized from the 6-well plates and washed in 10 ml of PBS three times by centrifugation (1000 r.p.m., 5 min), then the collected BMDMs were resuspended in 100 μl of PBS; 1 × 10^6^ polarized BMDMs were delivered to the remnant liver via portal vein injection by a 33G microsyringe (Hamilton, Reno, NV, USA) after hepatectomy. Postoperative mice were sacrificed at designated time points and the tissues were processed as mentioned above.

### Untargeted lipidomics

The conditional medium (CM) of polarized macrophages was collected at 48 h later after stimulation with LPS or IL-4, following by centrifuging at 1200 r.p.m. for 5 min to precipitate cell debris, and the supernatant was filtered by a 0.22-μm syringe filter and lyophilized by a freeze dryer. The lyophilized medium was used for lipid extraction. Tissues and cells were homogenized on ice in buffer [25 mM tris (hydroxymethyl)-aminomethane hydrochloride (Tris-HCl), pH 7.4, 150 mM NaCl, 1 mM ethylene diamine tetraacetic acid (EDTA), and 1 mM ethyleneglycoltetraacetic acid (EGTA)]. Lipid extraction was performed by using a modified Bligh and Dyer procedure as described previously^24^. Each individual lipid extract was reconstituted with a volume of 500 μl/mg of protein or lyophilized powder of 2 ml of the medium in 1:1 CHCl_3_/MeOH, and then flushed with nitrogen. Quality control samples were established by pooling the examined cell or medium samples. The samples were analyzed by a Thermo Fisher Scientific Vanquish Flex ultra-high-performance liquid chromatography (UHPLC) equipped with Thermo Fisher Scientific Orbitrap Fusion Tribrid High-Resolution Mass Spectrometer (Thermo Fisher Scientific, Waltham, MA, USA). Briefly, 5 μl of the lipid extract was injected, and chromatographic separation was carried out at 40 °C on an Acquity UPLC column C30 (150 × 2.1 mm, 3.0 µm). The mobile phase A was: 10 mM HCOONH_4_, 0.1% formic acid, acetonitrile (ACN):H_2_O = 60:40, mobile phase B: 10 mM HCOONH_4_, 0.1% formic acid, isopropanol (IPA):ACN = 90:10. The electrospray ionization source was used with both positive and negative ion modes. Mass scan were performed as the following settings: orbitrap resolution: 60,000; scan range: 200–2000 *m*/*z*; radiofrequency lens: 60%; automatic gain control target: 2.0e5; maximum injection time: 100 ms; polarity: positive. The identification of lipid molecular species was performed using Lipid Search software (Thermo Fisher Scientific; Waltham, MA, USA). These lipid-profiling data were then subjected to further analyses using the online Metaboanalyst tool (www.metaboanalyst.ca)^25^, the data first underwent a normalization using values of quality control samples, missing values for compounds that were not detected in all samples were excluded. Log2 transformation was conducted to create a heatmap. Log transformation was performed, and the data were autoscaled for principal component analysis (PCA) and orthogonal projections to latent structures discriminant analysis (OPLS-DA) analyses.

### Targeted lipidomics

The measurement of CER metabolites was performed according to the protocol reported by Wang et al.^26^. Tissues and cells were homogenized on ice in the buffer as aforementioned. Lipids from tissue homogenates (2 mg protein per sample) were extracted with ethyl acetate/isopropanol/water (60/30/10, v/v/v). Lipids from the medium of polarized macrophages (100 μl diluted with 1900 μl of serum-free RPMI medium) were extracted with ethyl acetate/isopropanol (85/15, v/v). The lipid extracts were dried under N_2_ gas stream and reconstituted in methanol, then CER, sphingosine (SPH), and S1P were determined by ultra-high-performance liquid chromatography–electrospray ionization mass spectrometry (UHPLC–ESI-MS/MS) performed on prelude SPLC + TSQ Quantiva LC-MS/MS system (Thermo Fisher Scientific, Waltham, MA, USA). Standards and internal standards of CER metabolites were purchased from Avanti Polar Lipids, Alabaster, AL, USA. Amounts of CER metabolites were quantified using standard curves of external standards, and then normalized to protein contents or medium volume.

### Immunohistochemistry

Polarized macrophages in liver were immunofluorescent double-labeled using M.O.M.^®^ (Mouse on Mouse) Basic Kit (Vector, Burlingame, CA, USA) according to the manufacturer’s instructions. Anti-F4/80 (Abcam, Cambridge, MA, USA) was used to stain macrophages. Anti-inducible nitric oxide synthase (iNos) antibody (BD, San Jose, CA, USA) and anti-CD206 antibody (Bio-Rad, Hercules, CA, USA) were used to label M1 and M2 macrophages, respectively. Alexa Fluor^®^ 488-conjugated antibody (Abcam, Cambridge, MA, USA), Alexa Fluor^®^ 594-conjugated antibody (Abcam, Cambridge, MA, USA), and Cy3-conjugated antibody (Vector, Burlingame, CA, USA) were used as secondary antibodies. For the liver tissues with GFP-transfected macrophages infusion, anti-GFP antibody (Abcam, Cambridge, MA, USA) and Alexa Fluor^®^ 488-conjugated secondary antibody were applied to detect GFP-positive macrophages. The stained sections and cells were mounted in an anti-fade solution with 4ʹ,6-diamidino-2-phenylindole (DAPI) (Abcam, Cambridge, MA, USA). Immunostaining on liver tissue sections was performed using VECTASTAIN^®^ Elite^®^ ABC HRP Kit (Vector, Burlingame, CA, USA) according to the manufacturer’s instructions, anti-proliferating cell nuclear antigen (PCNA) antibody (Cell Signaling Technology, Danvers, MA, USA) and anti-cleaved-caspase 3 (C-Caspase 3) antibody (Cell Signaling Technology, Danvers, MA, USA) were used to detect proliferation and apoptosis, respectively. Anti-hepatocyte nuclear factor 4α (HNF-4α) antibody (Abcam, Cambridge, MA, USA) was used to stain hepatocytes. Alexa Fluor^®^ 488-conjugated secondary antibody and Alexa Fluor^®^ 568-conjugated secondary antibody (Abcam, Cambridge, MA, USA) were used as secondary antibodies. Terminal-deoxynucleoitidyl transferase-mediated nick end labeling (TUNEL) assay on cells or liver sections was performed using a TdT In Situ Apoptosis Detection Kit (Minneapolis, MN, USA) according to manufacturer’s instructions. Stained cells and liver sections were observed, and positive cells were counted in five random fields under Intelligently Designed Microscope (Olympus, Shinjuku-ku, Tokyo, Japan) or LSM 880 with Airyscan Confocal Microscope (Zeiss, Oberkochen, Germany).

### Western blotting

Liver or cell homogenates were prepared in radioimmune precipitation buffer (RIPA) [50 mmol/L tris (hydroxymethyl)-aminomethane (Tris), 1% NP40, 0.25% deoxycholic acid sodium salt, 150 mmol/L NaCl, 1 mmol/L EGTA] containing 1 mM phenylmethanesulfonyl fluoride (PMSF) and a protease inhibitor cocktail (Roche, Indianapolis, IN, USA). Protein concentrations were measured with a BCA Protein Assay Kit (Invitrogen, Waltham, MA, USA) according to manufacturer’s manual. Thirty micrograms of protein extracts were denatured in Laemmli buffer containing 5% β-mercaptoethanol, then loaded and separated by gel electrophoresis on a 10% or 12% Bis-Tris gel. Primary antibodies were incubated at 4 °C overnight under shaking conditions. Immunoreactive bands were visualized on nitrocellulose filter membrane using horseradish peroxidase (HRP)-linked anti-mouse or anti-rabbit antibody (Cell Signaling Technology, Danvers, MA, USA) and the ECL Ultra Western HRP Substrate (Millipore, Burlington, MA, USA). Anti-PCNA antibody (Cell Signaling Technology, Danvers, MA, USA), anti-C-Caspase 3 antibody (Cell Signaling Technology, Danvers, MA, USA), and anti-cleaved PARP (C-PARP) antibody (Cell Signaling Technology, Danvers, MA, USA) were used to evaluate cell proliferation and apoptosis. The expression level of β-Actin (Abcam, Cambridge, MA, USA) was used as an internal control. Antibodies used in this study are listed in Supplemental Table 1.

### Quantitative reverse transcription PCR

Total RNA was extracted from cells or liver tissues using PureLink™ RNA Mini Kit (Invitrogen, Waltham, MA, USA) according to the manufacturer’s instructions. mRNA was reversely transcribed to cDNA by PrimeScript™ RT Master Mix (TaKaRa, Kusatsu, Shiga, Japan). Quantitative reverse transcription PCR (qPCR) was performed to determine the mRNA levels of M1-macrophage-associated genes, *Inos*, tumor necrosis factor-α (*Tnf-α*), and interleukin-6 (*IL-6*), M2-macrophage-associated genes, arginase1 (*Arg1*), inflammatory zone 1 (*Fizz1*), *Il-4*, and interleukin-10 (*Il-10*), and genes encoding CER metabolic enzymes. cDNA templates were diluted 1:5 and amplified with SYBR^®^ Premix Ex Taq™ II (TaKaRa, Kusatsu, Shiga, Japan) using Roche LightCycler^®^ 480 PCR System (Roche, Indianapolis, IN, USA). An initial denaturation at 95 °C for 3 min was followed with PCR cycling: 95 °C (5 s) and 60 °C (30 s) for 40 cycles. Relative mRNA levels were calculated by means of 2^−ΔΔCt^. *β-Actin* was used as the reference gene. Primer sequences used to amplify specific gene fragments are shown in Supplemental Table 2.

### Primary mouse hepatocytes and hepatic non-parenchymal cells isolation

Primary mouse hepatocytes were harvested from postoperative mice by a modified rapid two-step perfusion method^27^. Briefly, 48 h after hepatectomy, mice were retrogradely perfused from the inferior vena cava with Hank’s balanced salt solution (HBSS) and Dulbecco’s modified eagle medium (DMEM) containing collagenase IV (80 U/ml) (Worthington Industries, Columbus, OH, US) to digest liver tissues, and primary mouse hepatocytes were harvested from the digested liver by filtration through a 200-mesh filter followed by centrifugation at 500 × *g* for 2 min for three times. The remaining cells were resuspended in 30% Percoll followed by centrifugation at 450 × *g* for 20 min to remove cell debris; 25% and 50% stock isotonic Percoll (SIP) were prepared by mixing Percoll solution (GE Healthcare Life Sciences, Marlborough, MA, US) with PBS (Gibco, Waltham, MA, USA). A centrifuge tube containing 50% SIP solution (20 ml) and 25% SIP solution (20 ml) was prepared. Then the hepatic non-parenchymal cells (HNPCs) were added to the SIP of discontinuous isotonic gradient and centrifuged at 800 × *g* for 15 min. The HNPCs, including hepatic macrophages, were collected from the 25% SIP cushion^28^. After lysis of red blood cells, the HNPCs were washed and resuspended for fluorescence-activated cell sorting (FACS) analyses. Primary mouse hepatocytes were resuspended and cultured in DMEM for 24 h in 6-well plates and confocal dishes, then the medium was changed with 2 ml CM of polarized macrophages, or the medium was supplemented with CER metabolites, including10 μM d18:1/C_16_-CER (Avanti Polar Lipids, Alabaster, AL, USA) and 500 nM d18:1-S1P (Avanti Polar Lipids, Alabaster, AL, USA). After 48-h stimulation, hepatocytes were collected for immunostaining, protein extraction, and RNA isolation, as aforementioned.

### 3-(4,5-Dimethylthiazol-2-yl)-2,5 diphenyl tetrazolium assay

3-(4,5-Dimethylthiazol-2-yl)-2,5 diphenyl tetrazolium (MTT, Merck-Calbiochem, Burlington, MA, USA) was dissolved in Milli Q water (5 mg/ml). Primary mouse hepatocytes cultured in 12-well plates were incubated with a 300-μl mixture of MTT reagent (5 mg/ml) for 2 h. Then, 300 μl of lysis buffer (1 mM HCL and 10% Triton X-100 in isopropanol) was added, plates were incubated at room temperature and gently shaken at 70 r.p.m. for 30 min to lysate the cells and elute MTT dye. The absorbance was then measured at 590 nm used a spectrometer.

### FACS analysis

BMDMs or BMDMs with GFP-transfected were stained with PE-labeled anti-F4/80 (eBioscience, San Diego, CA, USA) or FITC-labeled anti-CD11b (eBioscience, San Diego, CA, USA). Prepared HNPCs were resuspended in PBS at 4 °C and pre-incubated with TruStain FcX™ (anti-mouse CD16/32) antibody (BioLegend Way, San Diego, CA, USA) to minimize non-specific antibody binding, then HNPCs were stained with PE-labeled anti-F4/80 antibody (eBioscience, San Diego, CA, US), FITC-labeled anti-CD11b antibody (eBioscience, San Diego, CA, US), and anti-GFP antibody (Abcam, Cambridge, MA, USA) with secondary antibody Alexa Fluor^®^ 647-conjugated antibody (Abcam, Cambridge, MA, USA). FACS analysis was performed using a FACS Calibur^TM^ flow cytometer (BD Immunocytometry Systems, San Jose, CA, USA). Data were analyzed with the FlowJo software version 10.0 (FlowJo, Ashland, OR, USA).

### Statistics

Data are expressed as the mean ± standard deviation (SD). Statistical analyses were performed using one-way analysis of variance (ANOVA) followed by Tukey’s post-hoc test or a Student’s *t*-test. *P* < 0.05 was considered statistically significant. Data analysis was performed using IBM SPSS Statistics for Windows version 22.0 (IBM; Armonk, NY, USA).

## Results

### Lipidomics reveals elevation of CER metabolites as signature lipid species relevant to M1/M2 polarization in BMDM

**Fig. 1:**
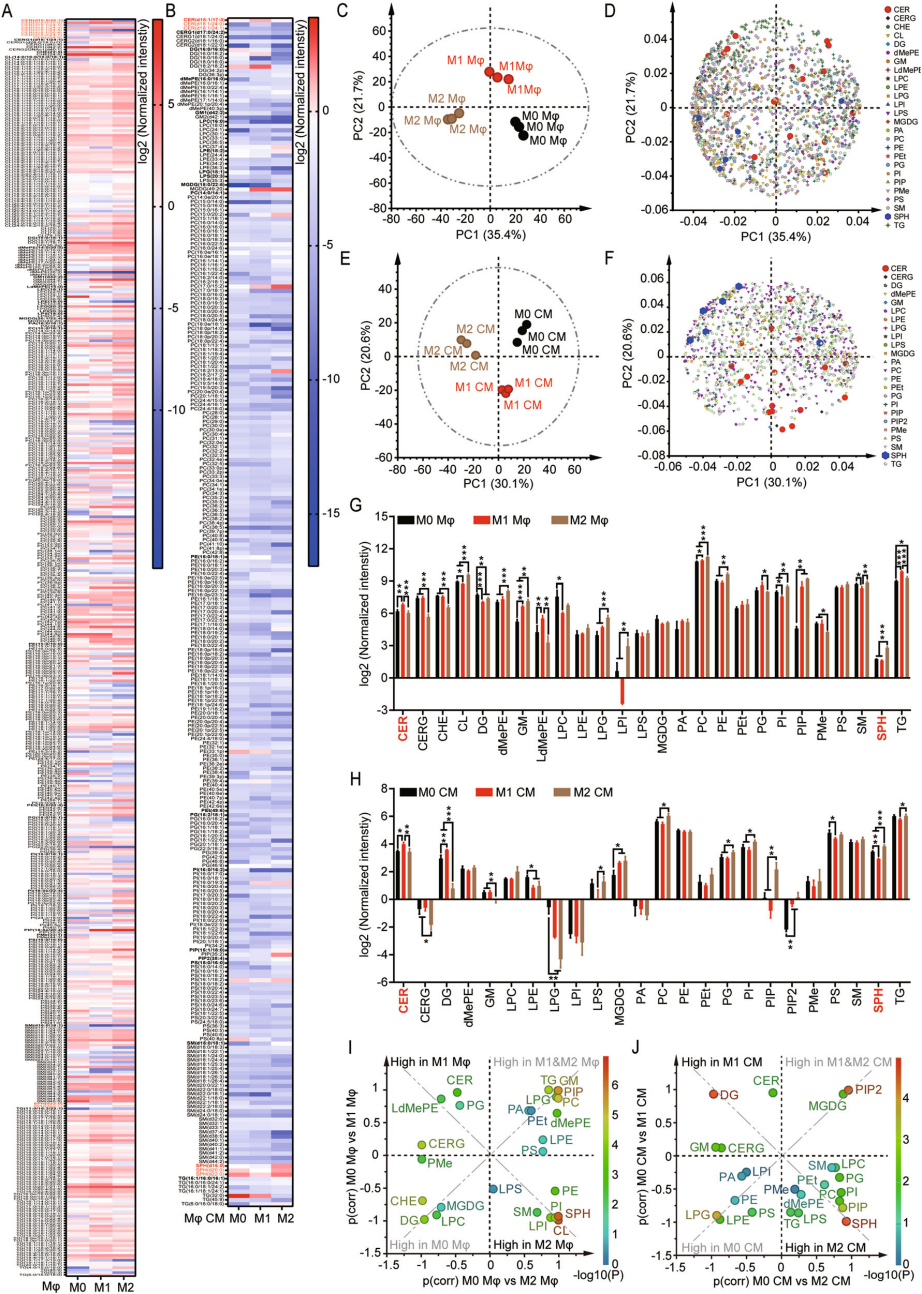
Untargeted lipidomics on polarized BMDMs. Mouse bone marrow cells were differentiated and polarized into M1 and M2 BMDMs by treatment of LPS and IL-4, respectively. Lipids were extracted from non-polarized and polarized BMDMs and their CM, then subjected to untargeted lipidomics analyses by UHPLC-ESI-MS/MS. **A**, **B** Content of individual lipid species in M0 or polarized BMDMs (**A**) and their CM (**B**). CER and SPH species were highlighted in red. **C**–**F** Lipid profiles of M0, M1, M2 BMDM cells and their CM were detected by untargeted lipidomics. PCA score plots and loading plots were used for exploratory data analysis of lipid dysregulation in BMDMs (**C** and **D**) and their CM (**E** and **F**) through dimensional reduction and data visualization. **G**, **H** Content of lipid classes in M0, M1, and M2 BMDMs (**C**) and their CM (**D**). **I**, **J** SUS-plot analysis based on orthogonal projections to OPLS-DA on lipid classes in polarized BMDMs (**I**) and their CM (**J**). Data represent results from three samples, each was pooled using BMDMs isolated from three mice. Data in **G** and **H** represent mean ± SD; *p < 0.05, **p < 0.01, ***p < 0.001.

Bioactive lipids produced by polarized macrophages have been implicated in mediating the distinct functions of polarized macrophages on tissue damage and repair^29^. In order to study lipids that have the potential to mediate the regulatory effects of polarized macrophages on liver injury and regeneration after hepatectomy, we first performed untargeted lipidomics analysis on polarized macrophages and their CM. Mouse bone marrow cells were isolated and differentiated into F4/80-positive BMDM cells (Supplementary Fig. 1A–C), then polarized to M1 BMDMs or M2 BMDMs by LPS or IL-4 stimulation, respectively (Supplementary Fig. 1D). Overall, a total of 1659 and 1251 individual lipid species were determined in the polarized macrophages and their CM, including lipid classes of CER, glycosyceramides (CERG), cholesterol ester (CHE), cardiolipins (CL), diglycerides (DG), dimethyiphosphatidylethanoiamines (dMePE), ganglioside (GM), lysodimethyiphosphatidylethanoiaminse (LdMePE), lysophosphatidylcholines (LPC), lysophosphatidylethanolamines (LPE), lysophosphatidylglycerols (LPG), lysophosphatidylinositols (LPI), lysophosphatidylserines (LPS), monogalactosyldiacylglycerol (MGDG), phosphatidic acid (PA), phosphatidylcholines (PC), phosphatidylethanolamines (PE), phosphatidylethanols (PEt), phosphatidylglycerols (PG), phosphatidylinositols (PI), phosphatidylinositol biphosphate (PIP_2_), phosphatidylinositol phosphate (PIP), phosphatidylmethanol (PMe), phosphatidylserines (PS), sphingomyelins (SM), SPH, and triglycerides (TG). In total, 514 and 281 individual lipid species were significantly altered in response to polarization in macrophages and their medium, respectively (Fig. 1A, B). PCA demonstrated the contents of individual lipid species in macrophages (Fig. 1C, D), and their CM (Fig. 1E, F) were clearly separated as classification of M1, M2, and M0. Notably, the levels of most detected CER species were found to be high in M1 macrophages (Fig. 1A, C, D). Whereas the CER-derived lipid metabolites, SPH, were high in M2 macrophages (Fig. 1B, E, F). To further highlight the distinct variations of lipid classes in polarized macrophages and their CM, we performed OPLS-DA (Supplementary Fig. 2) and shared and unique structure plot (SUS-plot) analysis on the content of lipid classes (Fig. 1C–F). The results revealed that the levels of CER, CERG, LdMePE, PG, DG, and GM were significantly upregulated in response to M1 polarization (Fig. 1G–J), among which, CERs were the most significantly increased lipid class in both M1 macrophages and their CM (Fig. 1I, J and Supplementary Fig. 2). On the other hand, among LPS, SM, PI, PE, and SPH that were significantly elevated in response to M2 polarization (Fig. 1G–J), the levels of CER-derived metabolites, SPH, were uncovered as the most significantly elevated lipid class in both M2 macrophages and their CM (Fig. 1I, J and Supplementary Fig. 2). These data identified CERs and their metabolites SPH as critical lipid signatures in differentiating M1 and M2 polarization in macrophages.

### M1 and M2 BMDMs highly express CER-synthetic and CER-catabolic pathways to produce CER and S1P, respectively

**Fig. 2:**
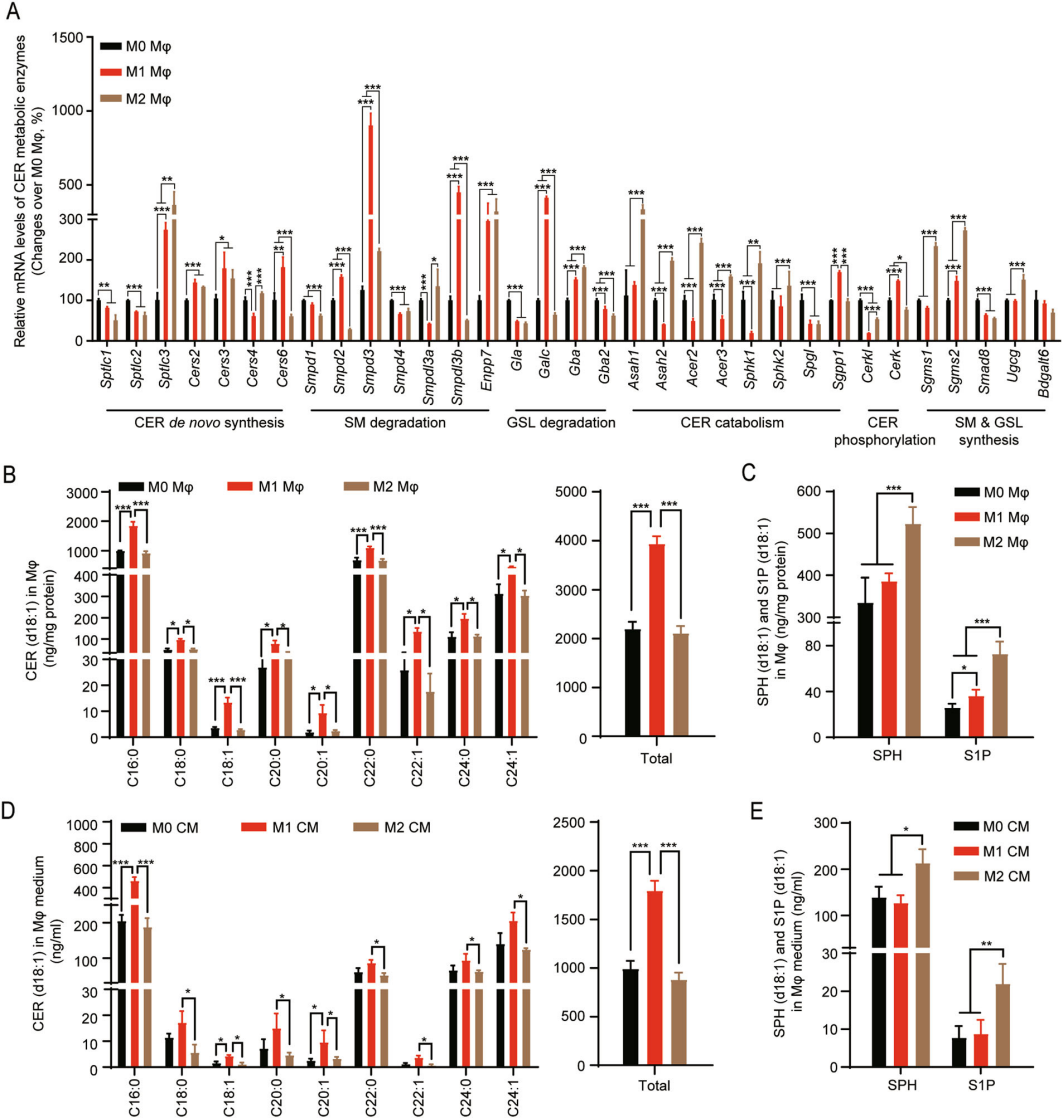
Polarized BMDMs express distinct CER metabolic pathway. **A** Total RNA of M0, M1, and M2 BMDMs was extracted to examine the mRNA levels of CER metabolic enzymes by qPCR. **B**–**E** Polarized BMDMs (**B** and **C**) and their CM (**D** and **E**) were collected to measure the levels of CER metabolites by targeted UHPLC-ESI-MS/MS, including d18:1-CER, d18:1-SPH, and d18:1-S1P. Data represent results from three independent experiments and demonstrated as mean ± SD; *p < 0.05, **p < 0.01, ***p < 0.001.

As we found that the levels of CER and their metabolites SPH were distinctly regulated in M1 and M2 BMDMs, we examined the difference in CER metabolic pathways in the polarized BMDMs by measuring the mRNA levels of genes encoding CER-metabolizing enzymes. We found that M1 macrophages expressed a CER-generation-metabolic pattern, which was characterized by upregulation of ceramide synthases (CERS) and hydrolase of SM and glycosphingolipids (GSL) that contribute to CER production, including *Cers2*, *Cers3*, *Cers6*, neutral sphingomyelinase 1 (*Smpd2*), neutral sphingomyelinase 2 (*Smpd3*), acid sphingomyelinase like 3A (*Smpdl3a*), and galactosylceramidase (*Galc*), and by downregulation of ceramidases (CDase) and sphingosine kinase (SPHK) which are involved in CER catabolism, including alkaline ceramidase 2 (*Acer2*), alkaline ceramidase 3 (*Acer3*), neutral ceramidase (*Asah2*), and *Sphk1* (Fig. 2A). However, M2 macrophages expressed a CER-catabolic pattern, which was characterized by dramatic upregulation of CDase and SPHK that hydrolyze CER, and synthase of SM and GSL that consume CER as substrates, including *Acer2, Acer3*, acid ceramidase (*Asah1*)*, Asah2, Sphk1*, sphingomyelin synthase 1 (*Sgms1*), sphingomyelin synthase 2 (*Sgms2*), and UDP-glucose ceramide glucosyltransferase (*Ugcg)* (Fig. 2A). S1P Lyase (*Sgpl*) that encoded the S1P hydrolase was downregulated in both M1 and M2 macrophages, S1P phosphatase 1 (*Sgpp1*) that catalyzes the dephosphorylation of S1P was downregulated in M1 macrophages (Fig. 2A).

Catabolism of CER is known to produce SPH, and SPH is then phosphorylated by SPHK to generate S1P. More importantly, S1P is currently found as a key signaling lipid that promotes hepatocyte proliferation and survival^19,20,30^. Since we found the mRNA levels of CDase and SPHK were dramatically upregulated by M2 polarization. We determined if S1P levels were consequently increased by M2 polarization. Therefore, we performed targeted lipidomics on d18:1-CER species, d18:1-SPH, and d18:1-S1P in polarized BMDMs and their CM. In accordance with the changes in metabolic enzymes, the levels of most d18:1-CER species were found to be significantly increased by M1 polarization (Fig. 2B–F), while the levels of d18-SPH and d18:1-S1P were significantly elevated in response to M2 polarization (Fig. 2B–F). These data collectively demonstrated that M1 macrophages expressed a CER-synthesis-dominant metabolism leading to an increase in CER accumulation, while M2 macrophages expressed a CER-catabolic metabolism resulting in upregulation of CER-derived S1P production.

### Infusion of M1 or M2 BMDMs correspondingly alters macrophage polarization and promotes CER or S1P generation in mouse liver after partial hepatectomy

**Fig. 3:**
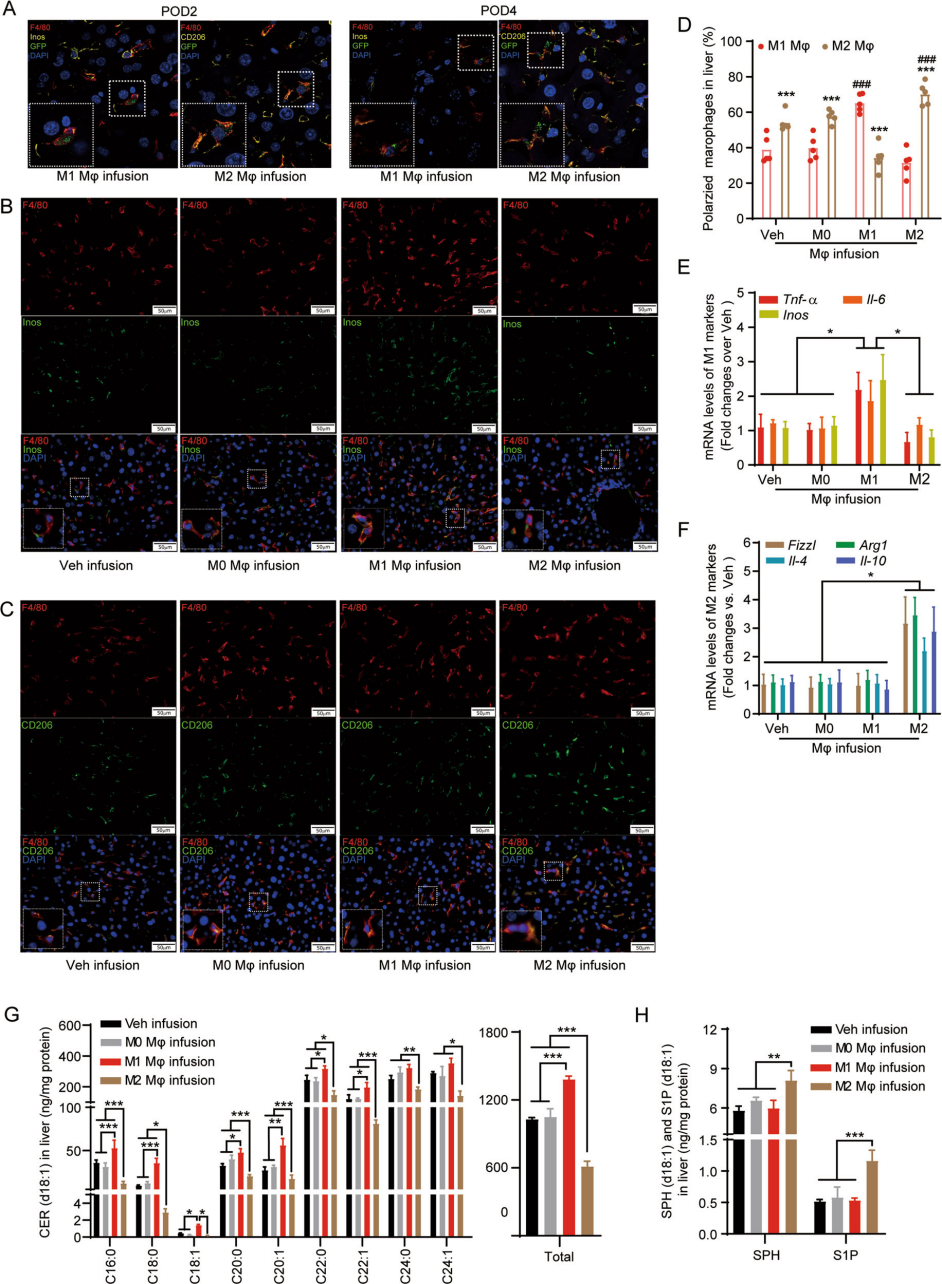
Infusion of ex vivo polarized BMDMs alters macrophage polarization and CER metabolism in the liver after partial hepatectomy. Mouse livers after hepatectomy were infused with phosphate buffer saline (PBS) (Veh), M0, M1, and M2 BMDMs. The remnant liver tissues were collected at postoperative day 2 (POD2) to examine macrophage polarization and levels of CER metabolites. **A** Liver tissues were harvested at 2 days and 4 days after GFP-positive polarized BMDM infusion. Liver sections were stained with anti-GFP (green), anti-F4/80 (macrophage marker, red), anti-Inos (M1 marker, yellow), and anti-CD206 antibodies (M2 marker, yellow) to validate the infiltration of polarized BMDM in the remnant liver after hepatectomy by confocal scanning. DAPI was used to visualize nuclei (blue). **B**, **C** Liver sections were co-stained with F4/80 (red) and iNos (green) (**B**) or CD206 (green) (**C**) to examine M1 or M2 macrophage polarization in response to BMDM infusion in the remnant liver after hepatectomy. DAPI was used to visualize nuclei (blue). **D** M1 and M2 macrophages in the remnant liver tissues were counted in five random 20× fields to quantify alteration in M1 and M2 polarization by BMDM infusion in the remnant liver after hepatectomy. **E**, **F** Total RNA was extracted from the remnant liver tissues, then mRNA levels of M1-macrophage-associated genes (*Tnf-α*, *Inos*, and *Il-6*) (**E**) and M2-macrophage-associated genes (*Fizzl*, *Arg1*, *Il-4*, and *Il-10*) (**F**) were determined by qPCR. **G**, **H** Levels of CER metabolites were determined in the remnant liver tissues with BMDM infusion after hepatectomy, including d18:1-CER (**G**), d18:1-SPH (**H**), and d18:1- S1P (**H**) by targeted UHPLC-ESI-MS/MS. Images in **A**–**C** represent results from five individual mice in each group. Data in **E**–**H** are demonstrated as mean ± SD, *n* = 5–6; *p < 0.05, **p < 0.01, ***p < 0.001.

Infusion of polarized macrophages has been applied to study the pathophysiological functions and cytotherapeutic potential of macrophage polarization^8^. To further investigate the functions of polarized macrophages in regulating hepatic CER metabolism and liver recovery after hepatectomy, artificially polarized BMDMs were infused into the remnant liver tissues after hepatectomy. The mouse model for studying post-hepatectomy liver injury and regeneration was established by 2/3 hepatectomy (Supplementary Fig. 3). A group of mice were first infused with BMDMs transfected with GFP, and both fluorescent microscopy and FACS analyses demonstrated that the GFP-transfected BMDMs indeed infiltrated into liver tissues after surgery (Supplementary Fig. 4). The polarized BMDMs were then infused into the remnant liver tissues of the donor’s littermate mice after hepatectomy. Confocal scanning validated that the exogenous M1 and M2 BMDMs indeed infiltrated into mouse liver and survived during liver recovery after hepatectomy (Fig. 3A). To investigate if the infusion of polarized BMDMs altered macrophage polarization in liver tissues, we examined the change in portions of polarized macrophages in the remnant liver after hepatectomy. We found that M1-BMDM and M2-BMDM injection correspondingly increased the portion of iNos-positive M1 macrophages and CD206-positive M2 macrophages (Fig. 3B–D). Simultaneously, M1-BMDM infusion was found to upregulate M1-associated genes in the remnant liver, including *Tnf-α*, *Il-6*, and *iNos* (Fig. 3E), whereas M2-BMDM infusion was found to elevate the expression of M2-associated genes in the remnant liver, including *Fizz1*, *Arg1*, *Il-4*, and *Il-10* (Fig. 3F).

Since M1 BMDMs and M2 BMDMs preferred producing CER and S1P, respectively, targeted lipidomics on d18:1-CER, d18:1-SPH, and d18:1-S1P were further performed to examine whether polarized BMDM infusion correspondingly increased the levels of CER and S1P in the remnant liver after hepatectomy. Indeed, we found that M1-BMDM infusion significantly increased the levels of most d18:1-CER species without affecting d18:1-SPH and d18:1-S1P (Fig. 3G, H). However, M2-BMDM infusion significantly reduced the levels of most d18:1-CER species and elevated the level of d18:1-SPH and d18:1-S1P in the remnant liver tissues (Fig. 3G, H). These data altogether demonstrated that infusion of M1 macrophages enhanced M1 polarization and promoted CER accumulation, while M2 macrophages infusion enhanced M2 polarization and promoting S1P production in the remnant liver after hepatectomy.

### Infusion of M1 and M2 BMDMs exerts distinct effects on promoting either injury or regeneration in mouse liver after partial hepatectomy

**Fig. 4:**
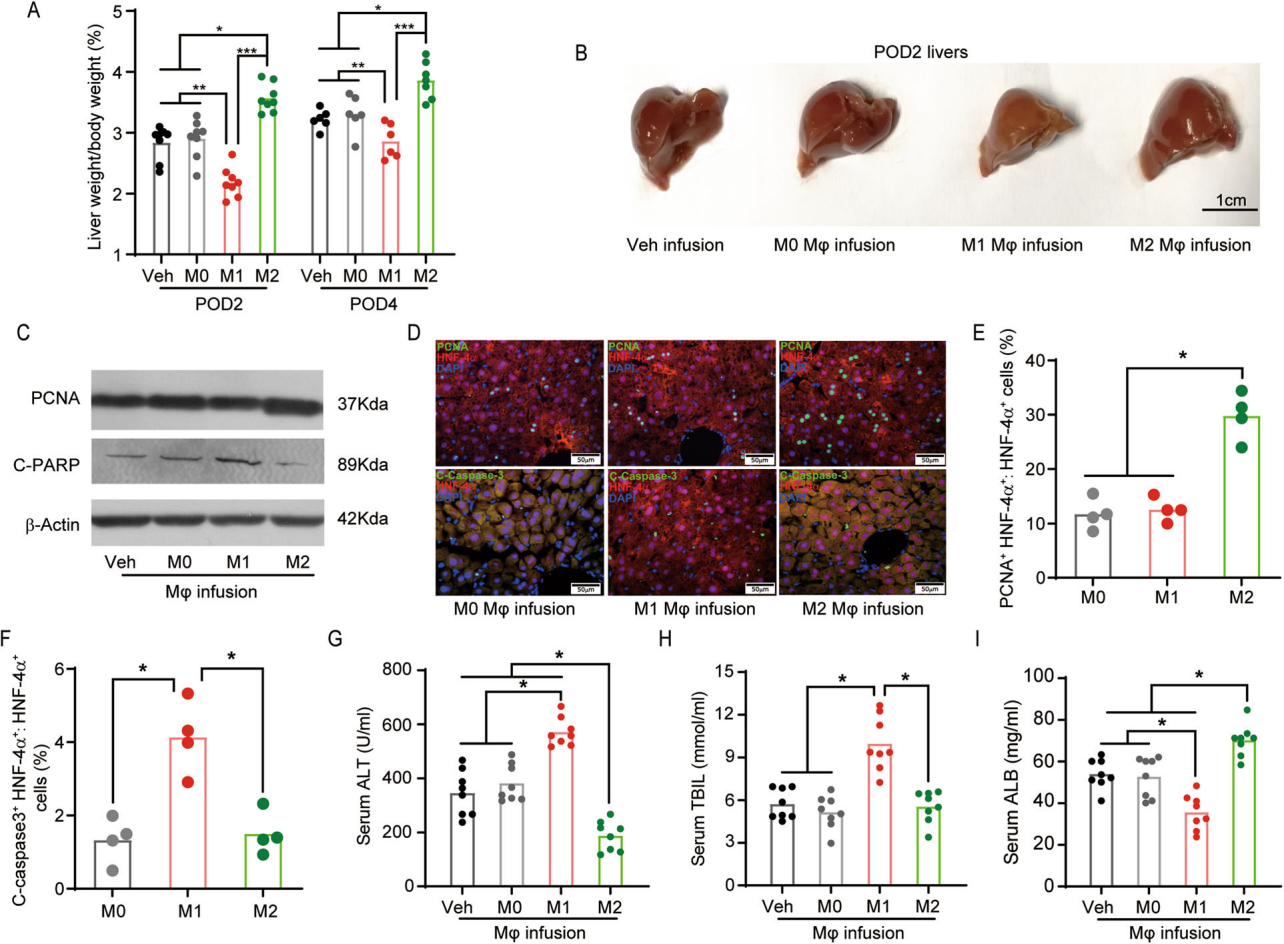
Infusion of M1 and M2 BMDMs exerts distinct effects on promoting either injury or regeneration in mouse liver after partial hepatectomy. Mice with infusion of polarized BMDMs were sacrificed at 2 days or 4 days after hepatectomy (POD2 or POD4), and the remnant liver tissues were collected to examine injury and regeneration. Remnant liver tissues collected at 2 days after hepatectomy were subjected to examination of proliferation and apoptosis. **A** Liver mass index (liver weight:body weight in %) was monitored after partial hepatectomy. **B** Representative images of the remnant liver after BMDM infusion. **C** Protein levels of proliferation marker PCNA and apoptosis marker C-PARP in the remnant livers were measured by immunoblotting. **D** Sections of liver tissues were co-stained with hepatocyte marker HNF-4α and proliferation marker PCNA or apoptosis marker C-Caspase 3. Top: representative images of staining of PCNA (green) and HNF-4α (red). Bottom: representative images of staining of C-Caspase 3 (green) and HNF-4α (red). Nuclei were counterstained with DAPI (blue). **E**, **F** The positive cells were counted, the percentage of PCNA^+^ and HNF-4α^+^ hepatocytes vs. HNF-4α^+^ cells (**E**) and the percentage of C-Caspase 3^+^ and HNF-4α^+^ hepatocytes vs. HNF-4α^+^ cells (**F**) were calculated. **G**–**I** Serum levels of ALT, TBIL, and ALB were determined at 2 days after surgery. Images in **B**–**D** represent results from five individual mice in each group; *p < 0.05, **p < 0.01, ***p < 0.001.

Since we found that the infusion of polarized BMDMs altered macrophage polarization and CER metabolites in mouse liver after hepatectomy, we further examined if the infusion of artificially polarized BMDMs affected liver injury and regeneration after hepatectomy. Liver regeneration index measurement revealed that M1-BMDM injection inhibited liver regeneration, while M2-BMDM infusion promoted liver regeneration (Fig. 4A). Morphological examination on the remnant livers demonstrated that M1-BMDM-infused mice had significantly smaller liver, while M2-BMDM-infused mice had larger liver (Fig. 4B). To examine the effects of BMDM infusion on liver regeneration and liver injury, the expression levels of proliferation marker and apoptotic maker in the liver tissues were measured. Immunoblotting demonstrated that M2-BMDM infusion elevated the proliferation marker PCNA and M1-BMDM infusion upregulated the apoptosis marker C-PARP (Fig. 4C). To further examine the hepatocyte-specific effects of BMDM infusion, HNF-4α antibody was used to label hepatocytes, then C-Caspase 3 and PCNA antibody were used to label apoptotic and proliferative cells, respectively. Immunofluorescent staining revealed that M2-BMDM infusion significantly increased the portion of PCNA/HNF-4α double-positive hepatocytes and the M1-BMDM infusion significantly increased the portion of C-caspase 3/HNF-4α double-positive hepatocytes (Fig. 4D–F), indicating that the M2-BMDM infusion indeed promoted hepatocellular proliferation and the M1-BMDM infusion worsened hepatocellular apoptosis in the hepatectomized liver. Liver function test further revealed that M1-BMDM infusion significantly elevated the levels of ALT and TBIL and reduced the levels of ALB, indicating M1-BMDM infusion aggravated post-hepatectomy liver dysfunction (Fig. 4H–J). M2-BMDM infusion significantly decreased the levels of ALT and ALB, but had no significant effect on TBIL levels (Fig. 4H–J). These data suggested that M1-BMDM infusion worsened hepatocyte apoptosis and aggravated liver dysfunction after hepatectomy, while M2-BMDM infusion promoted hepatocyte proliferation and attenuated post-hepatectomy liver dysfunction.

### CER and S1P mediate the functions of M1 and M2 BMDMs on regulating post-hepatectomy hepatocyte apoptosis and proliferation

**Fig. 5:**
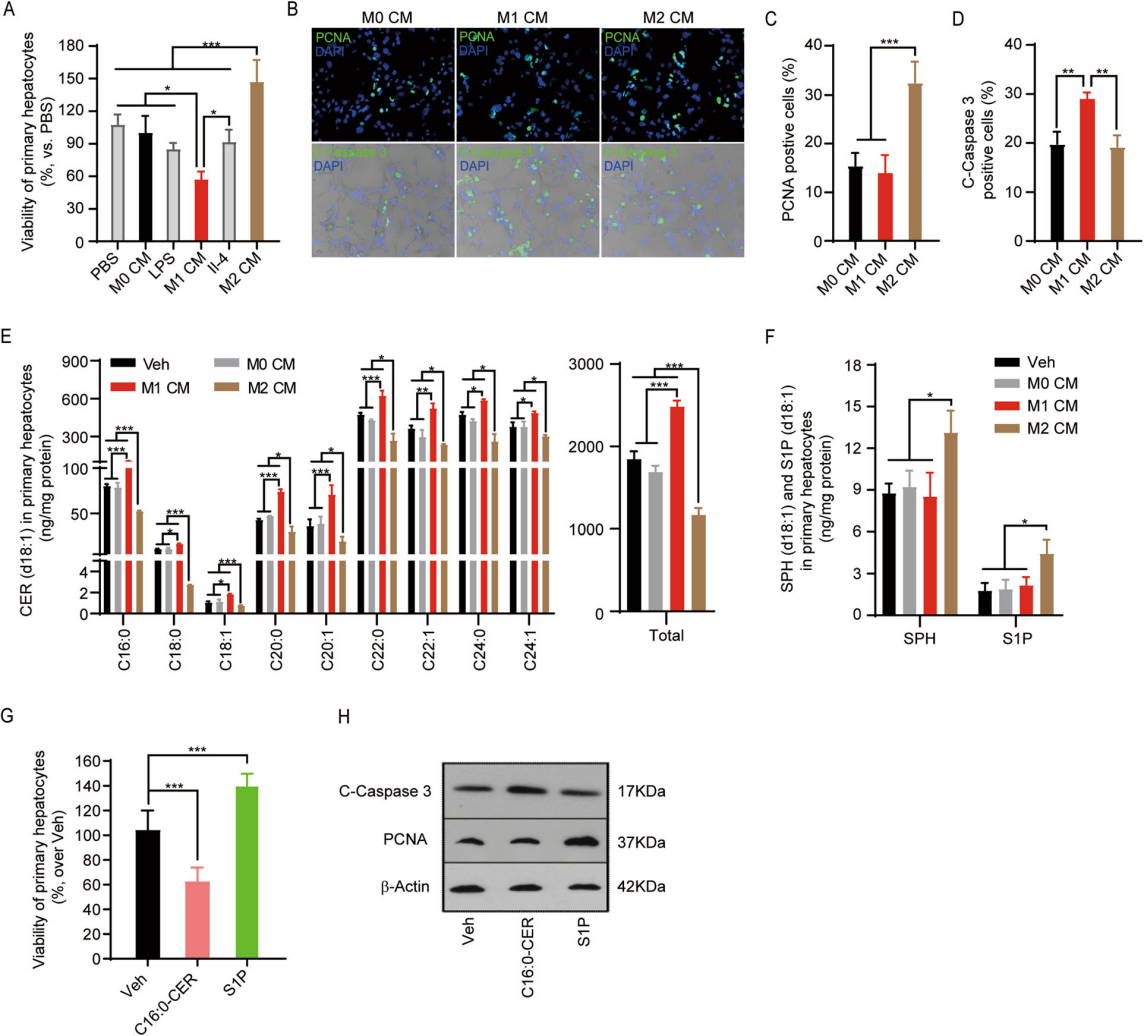
CER and S1P promote apoptosis and proliferation in post-hepatectomy hepatocytes, respectively. Primary hepatocytes were isolated from the remnant liver at 2 days after hepatectomy and incubated with CM from M0, M1, or M2 macrophages, or treated with 10 μM d18:1/C_16:0_-CER, 500 nM d18:1-S1P, 100 ng/ml LPS, and 10 ng/ml IL-4 for 48 h, then subjected to examination of proliferation and apoptosis. **A** The viability of hepatocytes treated with CM of BMDMs was measured by MTT assay. **B** Hepatocytes treated with CM of BMDMs were fixed and stained with proliferation marker PCNA and apoptosis maker C-Caspase 3. Top: representative images of immunofluorescent staining of PCNA (green). Bottom: representative images of immunofluorescent staining of C-Caspase 3 (green). Nuclei were counterstained with DAPI (blue). **C**, **D** PCNA-positive hepatocytes and C-Caspase 3-positive hepatocytes were enumerated to evaluate the proliferation (**C**) and apoptosis (**D**) in hepatocytes. **E**, **F** Hepatocytes treated with CM of BMDMs were collected to measure the levels of CER metabolites, including d18:1-CER (**E**), d18:1-SPH, and d18:1-S1P (**F**) by targeted UHPLC-ESI-MS/MS. **G** Primary hepatocytes isolated from post-hepatectomy mice were treated with d18:1/C_16:0_-CER (10 μm) and d18:1-S1P (500 nm) for 48 h, then the viability of treated cells was examined by MTT assays. **H** Protein levels of apoptosis marker C-caspase 3 and proliferation marker PCNA in d18:1/C_16:0_-CER (10 μm) and d18:1-S1P (500 nm) treated hepatocytes were measured by immunoblotting. Images in **B** and **H** represent results from three independent experiments. Data in **A** and **C**–**G** were demonstrated as mean ± SD, *n* = 3; *p < 0.05, **p < 0.01, ***p < 0.001.

Emerging studies implicate CER and S1P as essential bioactive lipids in regulating tissue injury and regeneration. In order to test if CER and S1P indeed mediate the distinct effects of BMDM infusion in promoting hepatocyte death and proliferation, we isolated mouse primary hepatocytes from the remnant liver after hepatectomy and then incubated them with the CM collected from BMDM or exogenous CER and S1P. MTT assay demonstrated that the viability of hepatocytes treated with the CM from M1 BMDMs was significantly reduced (Fig. 5A). However, hepatocyte viability was significantly increased by the treatment of M2-BMDM CM (Fig. 5A). The immunostaining results of apoptosis marker C-Caspase 3 and proliferation marker PCNA further revealed that M1-BMDM CM increased the number of C-Caspase 3 positive hepatocytes (Fig. 5B, C), but M2-BMDM CM elevated the number of PCNA-positive hepatocytes (Fig. 5B, D). These data indicate that M1-BMDM CM augments apoptosis, but M2-BMDM CM promotes proliferation in post-hepatectomy hepatocytes. Therefore, secretion from polarized macrophages indeed contributes to regulating proliferation and apoptosis in post-hepatectomy hepatocytes.

Next, we measured the levels of d18:1-CER, d18:1-SPH, and d18:1-S1P in primary hepatocytes incubated with the CM from polarized BMDMs. We found that M1-BMDM medium treatment significantly increased the levels of most d18:1-CER species in post-hepatectomy primary hepatocytes (Fig. 5E, F). However, treatment with M2-BMDM medium reduced the levels of most d18:1-CER species and elevated the levels of d18:1-SPH and d18:1-S1P in post-hepatectomy primary hepatocytes (Fig. 5E, F). To further evaluate the effects of CER and S1P on hepatocyte viability after hepatectomy, post-hepatectomy primary hepatocytes were treated with exogenous d18:1/C_16:0_-CER and d18:1-S1P. Examination of cell viability showed that d18:1/C_16:0_-CER reduced cell viability, whereas d18:1-S1P increased cell viability (Fig. 5G). Immunoblotting consistently demonstrated that d18:1/C16:0-CER treatment upregulated the protein levels of apoptosis marker C-Caspase 3 (Fig. 5H). However, d18:1-S1P increased the protein levels of proliferation marker PCNA in post-hepatectomy hepatocytes (Fig. 5H), suggesting that d18:1/C16:0-CER induced apoptosis, but d18:1-S1P promoted proliferation in post-hepatectomy hepatocytes.

**Fig. 6:**
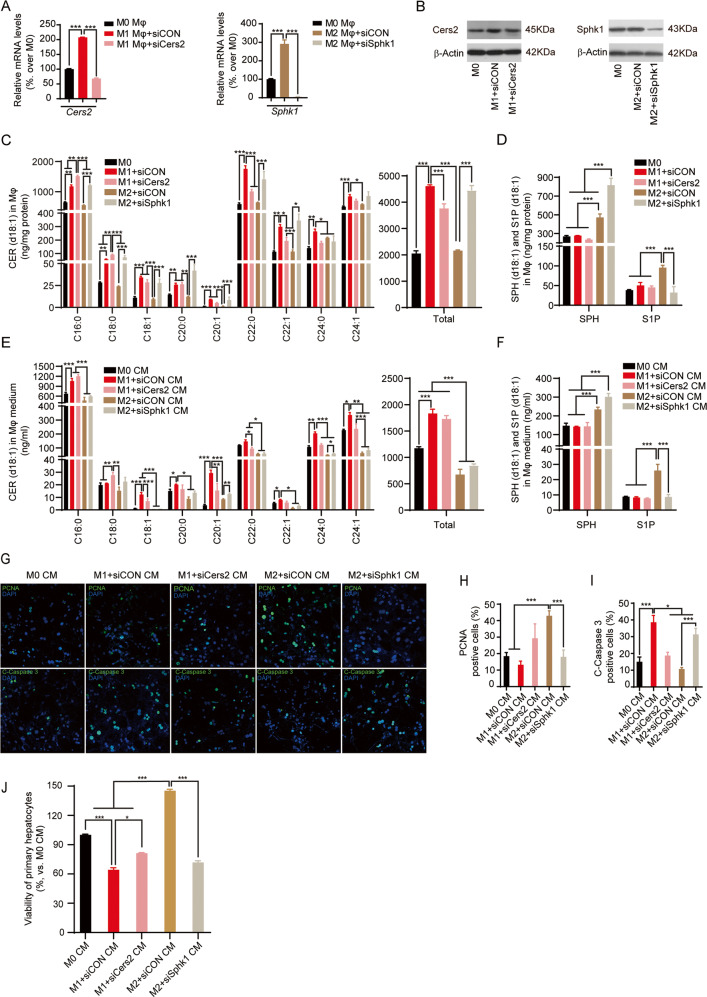
Inhibition of CER and S1P synthesis alters the M1- and M2-driven effects on post-hepatectomy hepatocytes, respectively. M1 BMDMs and M2 BMDMs were transfected with siRNA-Cers2 (siCers2) and siRNA-Sphk1 (siSphk1), respectively. A scramble siRNA was used as a control siRNA (siCON). Primary hepatocytes were isolated from the remnant liver at POD2 and incubated with CM from the transfected BMDM, then the proliferation and apoptosis of hepatocytes were examined. **A**, **B** Transfection was performed at 24 h before induction of polarization. mRNA (**A**) and protein (**B**) levels of Cers2 and Sphk1 were determined at 48 h after polarization. **C**–**F** BMDMs with siRNA transfection and their medium were collected to measure the levels of CER metabolites, including d18:1-CER (**C** and **E**), d18:1-SPH, and d18:1-S1P (**D** and **F**). **G** Hepatocytes were fixed and stained with proliferation marker PCNA and apoptosis maker C-Caspase 3. Top: representative images of immunofluorescence staining for PCNA (green). Bottom: representative images of immunofluorescence staining for C-Caspase 3 (green). Nuclei were counterstained with DAPI (blue). **H**, **I** PCNA-positive hepatocytes and C-Caspase 3-positive hepatocytes were enumerated to evaluate the proliferation (**H**) and apoptosis (**I**) in hepatocytes. **J** The viability of hepatocytes was measured by MTT assay. Images in **B** and **G** represent results from three independent experiments. Data in **A**, **C**–**F**, and **H**–**J** were demonstrated as mean ± SD, *n* = 3; *p < 0.05, **p < 0.01, ***p < 0.001.

To further verify that d18:1-CER and d18:1-S1P distinctly mediated the pro-death and pro-recovery effects of infused M1 and M2 BMDMs in post-hepatectomy liver. We knocked down Cers2 and Sphk1 in M1 and M2 BMDMs by siRNA transfection, respectively (Fig. 6A, B). Cers2 catalyzes the synthesis of long-chain CER^31,32^, and Cers2 was found to be upregulated in the M1 BMDMs (Fig. 2A). Sphk1 catalyzes the phosphorylation of SPH to produce S1P^33–35^, and Sphk1 was found to be upregulated in the M2 BMDMs (Fig. 2A). Consistently, targeted lipidomics demonstrated that CerS2 knockdown decreased the levels of long-chain CER species and total CER in M1 BMDMs and their CM, Sphk1 knockdown decreased the levels of S1P and accumulated SPH in M2 BMDMs and their CM (Fig. 6C–F). The post-hepatectomy primary hepatocytes were treated with the CM from these transfected BMDMs, and the proliferation and apoptosis of the hepatocytes were examined. Immunostaining revealed that Cers2 knockdown attenuated the pro-apoptotic effects of M1 CM, but Sphk1 knockdown endowed the M2 CM with pro-apoptotic effects instead of attenuating its pro-survival effects (Fig. 6G–I). Next, MTT assay showed that Cers2 knockdown attenuated the M1CM-induced decreased of cell viability (Fig. 6J). Notably, CM of Sphk1-knockdown M2 BMDMs reduced cell viability in hepatocytes (Fig. 6J).

These data collectively demonstrated that CER and S1P were critical lipids that mediated the effects of M1- and M2-BMDM infusion on promoting apoptosis and proliferation in post-hepatectomy hepatocytes, respectively.

## Discussion

Post-hepatectomy liver dysfunction caused by impaired liver recovery is a life-threatening morbidity, yet without efficient therapy^36^. Aiming to investigate the therapeutic capacity of polarized macrophages and the relevant lipid mediators for post-hepatectomy liver dysfunction, our study presented that CER and S1P were the critical signature lipids relevant to M1 and M2 polarization in macrophages. Moreover, our data from in vivo and in vitro studies demonstrated that CER and S1P critically mediated the hepatotoxic and hepatoprotective effects of M1- and M2-macrophages infusion on liver recovery after hepatectomy, respectively.

Emerging studies have implicated that bioactive lipids are important signatures of macrophages and play critical roles in mediating the functions of polarized macrophages^9,11,13^. Giannakis et al. reported that resolving D2, a lipid derived from polyunsaturated fatty acids, is a distinct signature of repair macrophages^9^. Montenegro-Burke et al. found that thromboxane A2 derived from arachidonic acid is a specific marker of M1 polarization^12^. In our study, we found that CERs were specifically elevated in response to M1 polarization, whereas SPH, the CER-derived lipid metabolites, were found to be increased by M2 polarization (Fig. 1 and Supplementary Fig. 2). With further investigation on the mRNA levels of CER enzymes in M1 and M2 BMDMs, our data further revealed a CER-producing-dominant metabolism in M1 macrophages and a CER-breakdown-dominant metabolism in M2 macrophages (Fig. 2A). Degradation of CER by CDase generates SPH, then SPH is phosphorylated by SPHK to produce S1P^37^. Thus, S1P is recognized as the final bioactive product of CER catabolism^37^. Consistent with the upregulation of CER catabolism in M2 BMDMs, our targeted lipidomics data further demonstrated that S1P was substantially elevated by M2 polarization (Fig. 2B–E). Intracellular CER, SPH, and S1P have been demonstrated to be secreted extracellularly through lipid vesicles or transporters^38,39^. Therefore, the intracellular increase of CER, SPH, and S1P in polarized macrophages possibly induces the extracellular secretion of CER, SPH, and S1P, resulting in elevation of these lipids in their CM.

Polarized macrophages have recently been implicated in regulating local lipid metabolism^29^. By injection of M1 BMDMs or M2 BMDMs into the remnant liver after hepatectomy, we found that in company with the corresponding alteration in macrophage polarization, M1-BMDM infusion also augmented the CER elevation in the remnant liver after hepatectomy, while M2-BMDM infusion reduced the levels of CER and increasing the levels of SPH and S1P (Fig. 3H, I). These data indicate that the infused polarized macrophages contribute to CER and S1P production in microenvironment of post-hepatectomy livers. Interestingly, consistently with in vivo experiments, by treating primary hepatocytes with the CM from polarized BMDMs, we also found that the CER levels or S1P levels were upregulated by the treatment of M1- or M2 CM (Fig. 5E, F). Despite CER and S1P produced by polarized BMDMs may directly enter liver cells to increase the intracellular levels of CER and S1P of liver cells, these data also suggest that polarized macrophages play a role in regulating the CER metabolism in targeted hepatocytes. Although the mechanism is still not clear, previous studies have found that the CER metabolic enzymes on the plasma membrane can be secreted within vesicles, then the vesicles containing CER metabolic enzymes can incorporate into targeted cells to regulate the CER metabolism^20^. Moreover, specific cytokines produced by polarized macrophages have also been found to regulate CER metabolism in targeted cells. For instance, TNF-α is known to upregulate CER synthesis^40^. Therefore, M1 and M2 macrophages may have the potential to directly or indirectly regulate CER metabolism in hepatocytes by incorporating their CERS, CDase, or SPHK into targeted hepatocytes or by producing cytokine through extracellular secretion.

CER and S1P are important bioactive lipids that distinctly regulate various cell biology related to tissue injury and regeneration^37^. CERs are reported as pro-apoptotic lipids. Both intracellular and extracellular accumulation of CER induces cell death by activating the particular apoptotic or necrotic pathway^41–43^. In contrast to CER, S1P is a pro-survival lipid that is crucial in promoting cell proliferation and survival^17,18^. Recent studies demonstrated that extracellular S1P, which was either stored in exosomes or bounded with high-density lipoprotein, was important to promote liver regeneration by enhancing angiogenesis and hepatocyte proliferation^19,20^. In line with these studies, we found that elevated hepatic CER induced by M1-BMDM infusion was associated with exaggerated hepatocyte injury after hepatectomy, while increased hepatic S1P induced by M2-BMDM infusion was found to improve liver regeneration after hepatectomy (Figs. 3 and 4). Furthermore, by treating the primary post-hepatectomy hepatocytes with exogenous d18:1/C_16:0_-CER and d18:1-S1P, we found that d18:1/C_16:0_-CER did induce apoptosis, whereas d18:1-S1P treatment promoted proliferation in post-hepatectomy hepatocytes (Fig. 5G, H). Besides bioactive lipids, cytokines produced by the polarized macrophages also play important roles in regulating tissue injury and recovery^44,45^, thus it was more likely that CER and S1P partially mediated the effects of polarized macrophages. We therefore inhibited CER and S1P synthesis in polarized BMDMs by knocking down relevant enzymes, then examined the alteration of M1- and M2-driven effects on post-hepatectomy hepatocytes. We found that knockdown of Cers2 inhibited the increase of CER in M1 BMDMs and their CM, and Cers2 knockdown was capable of partially attenuating the pro-death effect of M1 BMDMs (Fig. 6). These data suggested that inhibition of CER synthesis indeed partially attenuated the M1-driven effects. Due to the hydrophobic property of CER, the in vivo delivery system of CER has not yet been established, thus the direct impact of CER on post-hepatectomy hepatocytes has not yet been tested. Notably, we found that knockdown of Sphk1 inhibited the increase of S1P and accumulated SPH in M2 BMDMs and their CM, and the CM from Sphk1-knockdown BMDMs promoted cell death in mouse primary hepatocytes (Fig. 6). These data indicated that inhibition of S1P synthesis by Sphk1 knockdown endowed the M2 BMDMs with pro-apoptotic effects instead of attenuating its pro-survival effects, this might result from the accumulation of pro-apoptotic SPH^46^. Notably, consistent with our findings that S1P was important in driving liver recovery from hepatectomy, Nojima et al. have reported that injection of S1P-containing exosomes enhanced liver proliferation^20^. In addition to regulating hepatocyte survival, CER and S1P have also been implicated in regulating macrophage polarization. Our latest study demonstrated that the treatment of unsaturated-long-chain CER promoted the LPS-induced M1 activation on intraperitoneal macrophages^47^. On the other hand, S1P has been reported to promote M2 polarization by enhancing the production of anti-inflammatory cytokines, such as IL-4 and IL-10 (ref. ^48–50^). These results suggest that, in addition to regulating apoptosis and proliferation in hepatocytes after hepatectomy, elevation of CER and S1P caused by M1- and M2-BMDM infusion may also involve in driving M1 and M2 polarization of macrophages in the remnant liver after hepatectomy, respectively.

## Conclusions

Our study demonstrates for the first time that CER metabolites are important lipid mediators for the polarized macrophages to regulate liver injury and regeneration after hepatectomy. Particularly, our data highlight that M2 macrophage infusion that promotes the generation of hepatoprotective S1P may have cytotherapeutic capacity for post-hepatectomy liver dysfunction.

## Supplementary information

Supplementary Figure 1

Supplementary Figure 2

Supplementary Figure 3

Supplementary Figure 4

Supplemental Information

Supplemental Table 1

Supplemental Table 2
