# Supplementary figures and images for: Ceramides and sphingosine-1-phosphate mediate the distinct effects of M1/M2-macrophage infusion on liver recovery after hepatectomy
Source: Cell Death Dis. 2021 Mar 26;12(4):324. doi: 10.1038/s41419-021-03616-9 (PMC7998020; doi:10.1038/s41419-021-03616-9)

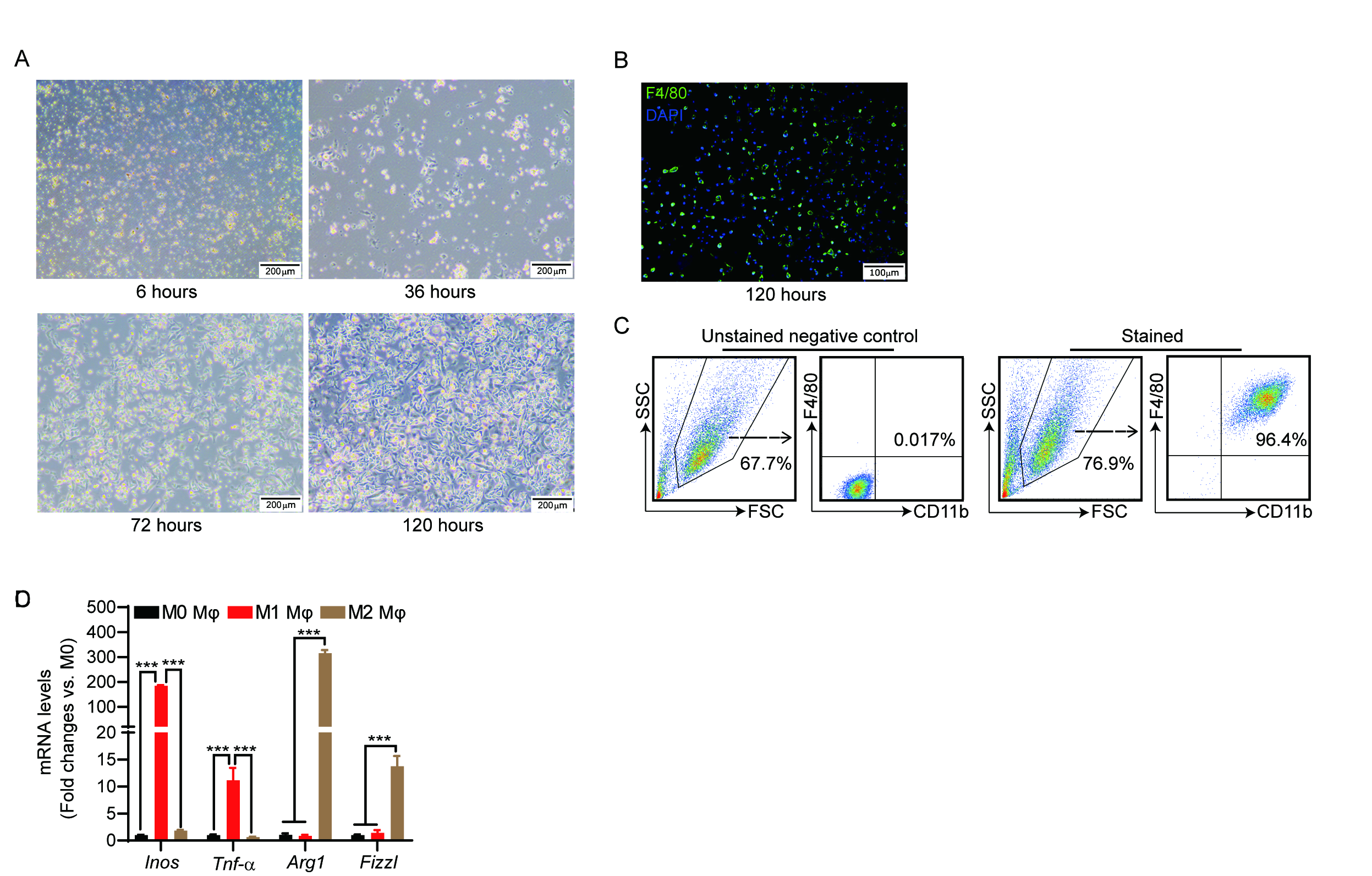

Supplement: Supplementary file 1 — Supplementary Figure 1 [file 41419_2021_3616_MOESM1_ESM.tif]

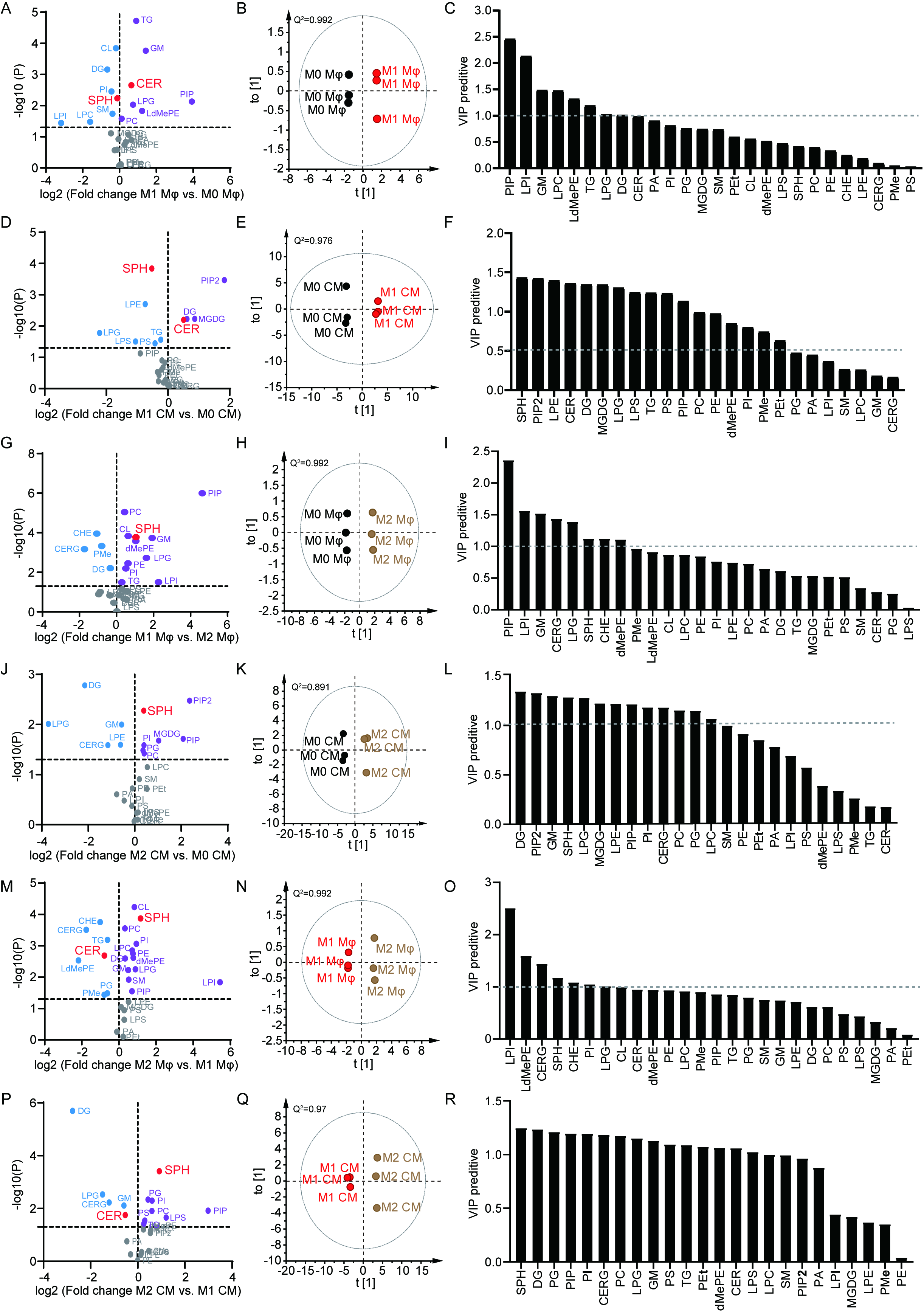

Supplement: Supplementary file 2 — Supplementary Figure 2 [file 41419_2021_3616_MOESM2_ESM.tif]

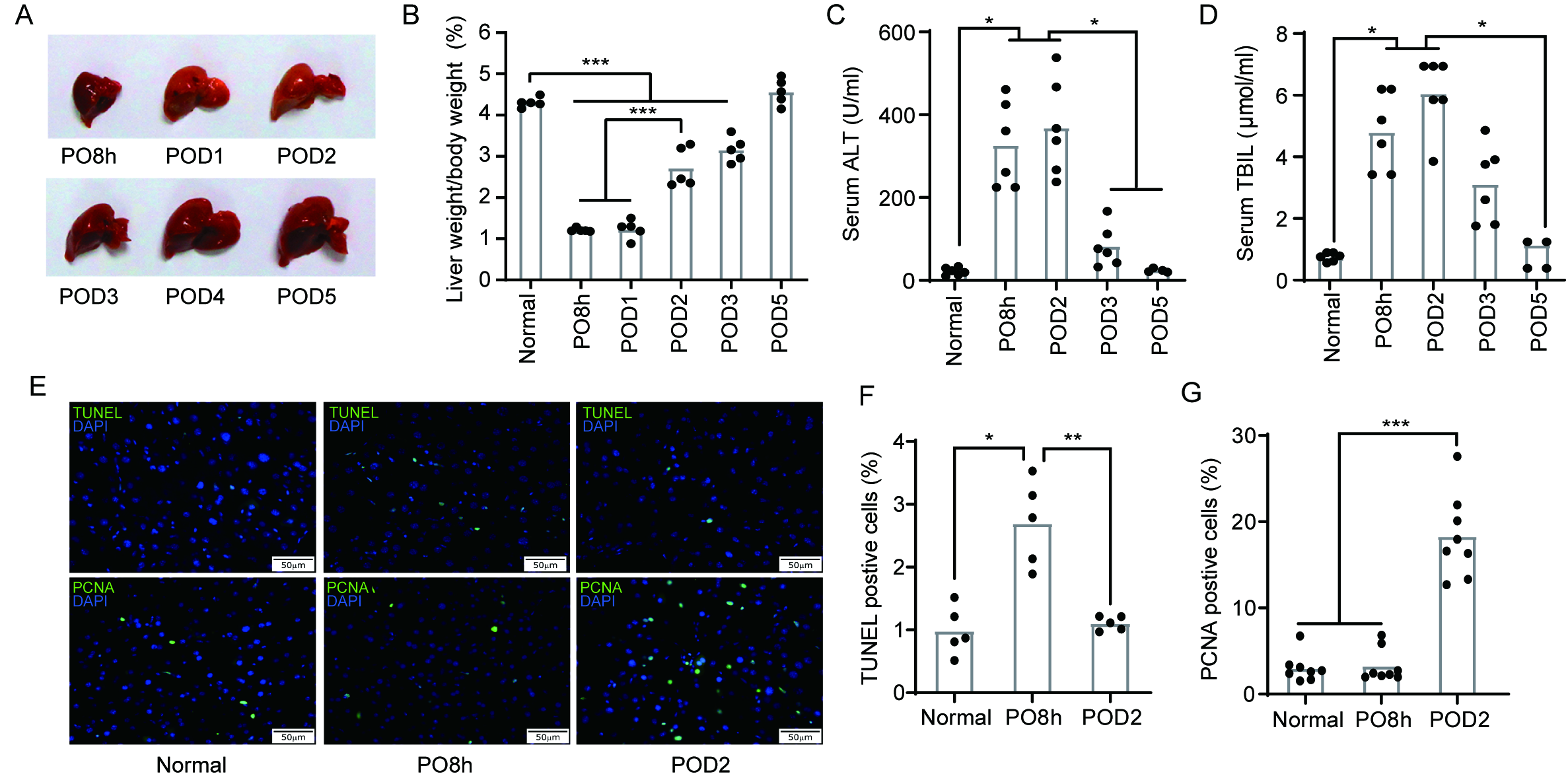

Supplement: Supplementary file 3 — Supplementary Figure 3 [file 41419_2021_3616_MOESM3_ESM.tif]

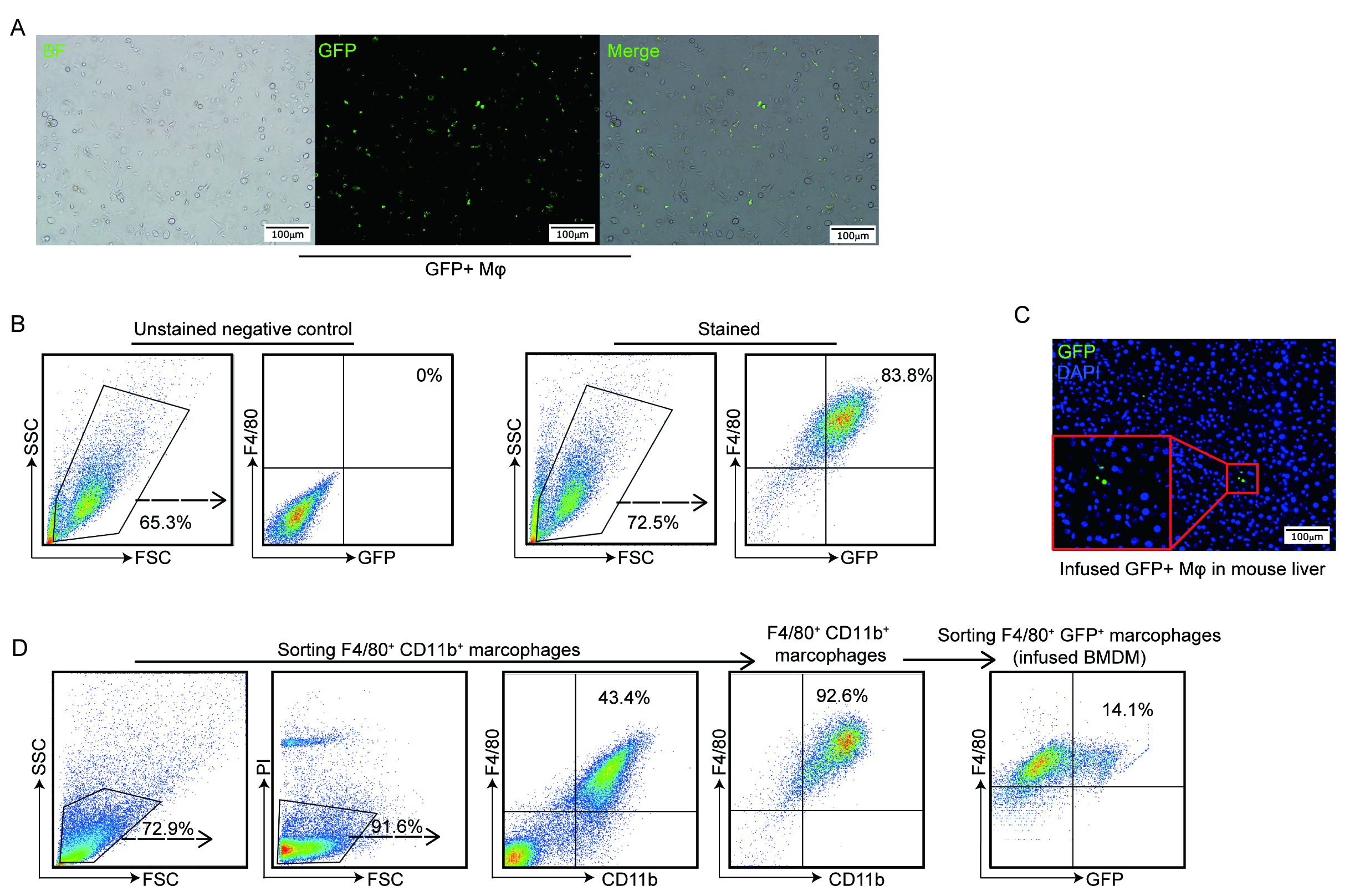

Supplement: Supplementary file 4 — Supplementary Figure 4 [file 41419_2021_3616_MOESM4_ESM.tif]
