# Supplementary material for: Ceramides and sphingosine-1-phosphate mediate the distinct effects of M1/M2-macrophage infusion on liver recovery after hepatectomy
Source: Cell Death Dis. 2021 Mar 26;12(4):324. doi: 10.1038/s41419-021-03616-9 (PMC7998020; doi:10.1038/s41419-021-03616-9)
Supplement: Supplementary file 5 — Supplemental Information [file 41419_2021_3616_MOESM5_ESM.docx]

**Supplemental Figure Legends**

**Figure S1. Bone-marrow-derived macrophages (BMDM) culture and polarization.**

(**A** and **B**) Mouse bone marrow cells were isolated and differentiated into BMDM (**A**). F4/80 (green) staining was applied to examine the differentiation of BMDM (**B**). DAPI was used to visualize nuclei (blue). The percentage of F4/80 positive cells in (B) was 97%. (**C**) Differentiated BMDM were analyzed by FACS after staining with PE-labeled anti-F4/80 and FITC-labeled anti-CD11b antibodies. The unstained cells were used as a negative control for gating the positive cells. (**D**) BMDM were polarized into M1 BMDM and M2 BMDM by LPS and Il-4 stimulation, respectively. Total RNA was extracted from polarized BMDM, and M1 signature genes (*Inos* and *Tnf-α*) or M2 signature genes (*Arg1* and *Fizzl*) were determined by qPCR. Images and data in **A** to **C** represent results from 3 independent experiments, Data in **D** are demonstrated as mean ± SD, n=3. ****p<*0.001.

**Figure S2. Volcano plots and** **OPLS-DA of lipid classes in** **polarized BMDM and their CM**. The significant compounds ranked by VIP scores. n=3, each sample was pooled using BMDM isolated from 3 mice.

**Figure S3. The process of liver recovery after partial hepatectomy.**

Mice received 2/3 liver resection were sacrificed at designated time points. The remnant liver tissues were collected for histologic analyses. (**A**) Changes in liver size at designated time points after partial hepatectomy were demonstrated in image **A**. (**B**) Liver regeneration was monitored and illustrated as a percentage ratio of liver weight and body weight. (**C** and **D**) Liver dysfunction was assessed by determining the levels of serum ALT (**C**) and TBIL (**D**) at designated time points after hepatectomy. (**E**-**G**) Liver sections were stained by TUNEL (green) to evaluate hepatocyte apoptosis and by PCNA (green) to evaluate hepatocyte proliferation (**E**). Cell nuclei were stained with DAPI (blue). Apoptotic cells (**F**) and proliferating cells (**G**) were counted in 5 random 20 × fields. Images in **A** and **E** represent results from 5-6 individual mice at each time point. **p*<0.05, ***p<*0.01, ****p<*0.001.

**Figure S4. Infusion of GFP-transfected BMDM in mouse liver after partial hepatectomy.**

(**A** and **B**) Mouse BMDM were transfected with a plasmid expressing GFP. Fluorescent microscopy (**A**) and FACS analyses (**B**) validated the expression of GFP in BMDM. The unstained cells were used as a negative control for gating the positive cells. (**C**) GFP-positive BMDM were infused into the hepatectomized liver, then liver sections were prepared at 48 hours after surgery to observed GFP-positive BMDM under fluorescent microscopy. (**D**) After GFP-positive BMDM infusion, the non-parenchymal cells (HNPCs) that contained all the macrophages in the mouse liver were isolated at 48 hours after hepatectomy. The HNPCs were labeled with anti-F4/80, anti-CD11b, and anti-GFP antibodies. The number of dead cells was measured by propidium iodide (PI) staining. The F4/80 and CD11b double-positive macrophages were sorted by FACS using the gating as that in Figure S1C. Then the GFP-positive macrophages, which were the infused BMDM, in these F4/80 and CD11b double-positive macrophages were sorted by FACS using the GFP gating as that in Figure S4B. Images represent results from 3 independent experiments.
