# Supplementary material for: Ceramides and sphingosine-1-phosphate mediate the distinct effects of M1/M2-macrophage infusion on liver recovery after hepatectomy
Source: Cell Death Dis. 2021 Mar 26;12(4):324. doi: 10.1038/s41419-021-03616-9 (PMC7998020; doi:10.1038/s41419-021-03616-9)
Supplement: Supplementary file 6 — Supplemental Table 1 [file 41419_2021_3616_MOESM6_ESM.docx]

**Table 1: Antibodies used in this study**

| **Name** | **Species** | **Supplier** | **Cat no.** | **Clone no.** |
| --- | --- | --- | --- | --- |
| F4/80 | rat | Abcam | ab6640 | CI: A3-1 |
| GFP | rabbit | Abcam | ab183734 | EPR14104 |
| PCNA | rabbit | CST | 13110 | D3H8P |
| Ki67 | rabbit | CST | 12075 | D3B5 |
| Cleaved PARP | rabbit | CST | 9532 | 46D11 |
| Cleaved-Caspase 3 | rabbit | CST | 9664 | Asp175 |
| iNos | mouse | BD | 610328 |  |
| CD206 | mouse | BIO-RAD | MCA2235 | MR5D3 |
| β-Actin | rabbit | Bioworld | AP0060 |  |
| anti-rabbit IgG HRP |  | CST | 7074 |  |
| Cy3 anti-rat IgG |  | VECTOR | CY-4300 |  |
| Alexa Fluor 594 anti-mouse IgG |  | Abcam | ab150116 |  |
| Alexa Fluor488 anti-rabbit IgG |  | Abcam | ab150077 |  |
| Alexa Fluor488 anti-mouse IgG |  | Abcam | ab150113 |  |
| HNF-4α |  | Abcam | ab41898 |  |
| PE-labeled anti-F4/80 | mouse | eBioscience | 12-4801-82 |  |
| FITC-labeled anti-CD11b | mouse | eBioscience | 17-2061-80 |  |
| anti-mouse CD16/32 | mouse | BioLegend | 101319 |  |
