# Supplementary material for: Ceramides and sphingosine-1-phosphate mediate the distinct effects of M1/M2-macrophage infusion on liver recovery after hepatectomy
Source: Cell Death Dis. 2021 Mar 26;12(4):324. doi: 10.1038/s41419-021-03616-9 (PMC7998020; doi:10.1038/s41419-021-03616-9)
Supplement: Supplementary file 7 — Supplemental Table 2 [file 41419_2021_3616_MOESM7_ESM.docx]

**Table 2: QPCR primers and corresponding sequences.**

| Name | Primer Sequence | Supplier |
| --- | --- | --- |
| *β-Actin F* | 5’-CTGAATGGCCCAGGTCTGA-3’ | Sangon Biotech Co., Ltd., Shanghai, China |
| *β-Actin R* | 5’-CCCTGGCTGCCTCAACAC-3’ |  |
| *Inos F* | 5’-AATCTTGGAGCGAGTTGTGG-3’ |  |
| *Inos R* | 5’-CAGGAAGTAGGTGAGGGCTTG-3’ |  |
| *Tnf-α F* | 5’-CATCTTCTCAAAATTCGAGTGACAA-3’ |  |
| *Tnf-α R* | 5’-TGGGAGTAGACAAGGTACAACCC-3’ |  |
| *Arg1 F* | 5’-CTCCAAGCCAAAGTCCTTAGAG-3’ |  |
| *Arg1 R* | 5’-AGGAGCTGTCATTAGGGACATC-3’ |  |
| *Fizz1 F* | 5’-TCCCAGTGAATACTGATGAGA-3’ |  |
| *Fizz1 R* | 5’-CCACTCTGGATCTCCCAAGA-3’ |  |
| *Il-4 F* | 5’-AGATGGATGTGCCAAACGTCCTCA-3’ |  |
| *Il-4 R* | 5’-AATATGCGAAGCACCTTGGAAGCC-3’ |  |
| *Cers2 F* | 5’-TCATCCCTTCTCAGTATTGGT-3’ |  |
| *Cers2 R* | 5’-ATCCTTTCGCTTGACATCAG-3’ |  |
| *Cers3 F* | 5’-GGCGATTTACATTTTACTTGCTG-3’ |  |
| *Cers3 R* | 5’-GGTCATATGCCCATGGTTTG-3’ |  |
| *Cers6 F* | 5’-TGTGCCATAGCCCTCAAC-3’ |  |
| *Cers6 R* | 5’-CTCCGAACATCCCAGTCC-3’ |  |
| *Smpd2 F* | 5’-GCCCAGTTCATCCACCAC-3’ |  |
| *Smpd2 R* | 5’-CCTCAGTCTCAACGAAAGC-3’ |  |
| *Smpd3 F* | 5’-TCATGGACGTGGCCTATC-3’ |  |
| *Smpd3 R* | 5’-ACCTGCACCTTGAGAAACAG-3’ |  |
| *Smpdl3a F* | 5’-CCTTTGCTGCCTACTGGTT-3’ |  |
| *Smpdl3a R* | 5’-ATTTGCGCCTTTAGATGAA-3’ |  |
| *Acer2 F* | 5’-GTGTGGCATATTCTCATCTG-3’ |  |
| *Acer2 R* | 5’-TAAGGGACACCAATAAAAGC-3’ |  |
| *Acer3 F* | 5’-GATTCACTGAGGAACTTTCG-3’ |  |
| *Acer3 R* | 5’-AGAGAAACTTCACTTTTGGC-3’ |  |
| *Aash1 F* | 5’-AATAACACTTGGGTTGTCAC-3’ |  |
| *Aash1 R* | 5’-TAGGATACCCAGATAACCAC-3’ |  |
| *Aash2 F* | 5’-AGAGAGAGCAAGGTATTCTTC-3’ |  |
| *Aash2 R* | 5’-ACTATTCACAAAGTGGTTGC-3’ |  |
| *Sphk1 F* | 5’-ACAGACCATCCAAAGGTAGTTT-3’ |  |
| *Sphk1 R* | 5’-CTCTATTCTGTGCTCAGTCTGTC-3’ |  |
| *Sgms1 F* | 5’-GGTCGTCCATGAACGAGTA-3’ |  |
| *Sgms1 R* | 5’-TGAAATAGCCAGAGTCCTACAA-3’ |  |
| *Sgms2 F* | 5’-TGGTATTGGTTGGGTTATGG-3’ |  |
| *Sgms2 R* | 5’-CGGGCACAGGTAACGTAGTG-3’ |  |
| *Ugcg F* | 5’-GCTTCGTGCTCTTCGTGG-3’ |  |
| *Ugcg R* | 5’-TTGCCTTCTTGTTGAGGTGT-3’ |  |
| *Sgpl F* | 5’-GAACCGACCTCCTCAAGCT-3’ |  |
| *Sgpl R* | 5’-CCCTGGCTGCCTCAACAC-3’ |  |
| *Sgpp1 F* | 5’-GAGCAACTTGCCGCTCTACTA-3’ |  |
| *Sgpp1 R* | 5’-GGTCGAGATTCCAGATCCAGAA-3’ |  |
| *Il-6 F* | 5’-AAGAAATGATGGATGCTACC-3’ |  |
| *Il-6 R* | 5’-GAGTTTCTGTATCTCTCTGAAG-3’ |  |
| *Sptlc1 F* | 5’-TACGAGGCTCCAGCATACC-3’ |  |
| *Sptlc1 R* | 5’-TCAGAACGCTCCTGCAACT-3’ |  |
| *Sptlc2 F* | 5’-CCATGCGTCACTGGTTCTA-3’ |  |
| *Sptlc2 R* | 5’-GTCCGAGGCTGACCATAAA-3’ |  |
| *Sptlc3 F* | 5’-ACATCCATGAGTCCCGTAG-3’ |  |
| *Sptlc3 R* | 5’-TCCATACCTCCAATGTTCC-3’ |  |
| *Cers4 F* | 5’-ACCCTGAATTTGTCCCTGTA-3’ |  |
| *Cers4 R* | 5’-CTTGAAGTCCTTGCGTTTG-3’ |  |
| *Smpd1 F* | 5’-GCCTGCAAAGTCTTATTCAC-3’ |  |
| *Smpd1 R* | 5’-CACCACATCGTCCTCAAAG-3’ |  |
| *Smpd4 F* | 5’-GGAATCTCCGATGCCTACA-3’ |  |
| *Smpd4 R* | 5’-ATCATTGGACCACTTGGGT-3’ |  |
| *Smpdl3b F* | 5’-TATCAACTCGTCCTTGTATGCC-3’ |  |
| *Smpdl3b R* | 5’-GAGGTTGGTCAAGCGTTCC-3’ |  |
| *Enpp7 F* | 5’-CGGCAAATACATCGAGAACC-3’ |  |
| *Enpp7 R* | 5’-CTCTGGATACCGAGCGTGGC-3’ |  |
| *Gla F* | 5’-ACCCTTTCATAAGCCCAATT-3’ |  |
| *Gla R* | 5’-GGTCCAGCGACTTCAACAA-3’ |  |
| *Galc F* | 5’-CCGATTTCCTCTTCCTTGCT-3’ |  |
| *Galc R* | 5’-GGTTCAATATGCGACTCCAA-3’ |  |
| *Gba F* | 5’-TCGTGTTAAACCGATCTTCG-3’ |  |
| *Gba R* | 5’-TGAATGGAGTAGCCAGGTGA-3’ |  |
| *Gba2 F* | 5’-CGTCCTTTGCCCTCGTC-3’ |  |
| *Gba2 R* | 5’-TGCCACCACTCCACTCATC-3’ |  |
| *Sphk2 F* | 5’-GTACTCATGTTGGGCATCTT-3’ |  |
| *Sphk2 R* | 5’-CATACTCCACTAACTCCCCA-3’ |  |
| *Cerkl F* | 5’-GAAGCATGGCTCTTAGGGT-3’ |  |
| *Cerkl R* | 5’-CTCCTCCTGTGGGCTGTAT-3’ |  |
| *Cerk F* | 5’-ATCTCCACGGGACAATAAA -3’ |  |
| *Cerk R* | 5’-GGCCATACAGGGCTTTC-3’ |  |
| *Samd8 F* | 5’-CAGACCTACCCACCACTCC-3’ |  |
| *Samd8 R* | 5’-TAGCACAGAATCACGCCAC-3’ |  |
| *B4galt6 F* | 5’-AAACAGCGGCTGGAATT-3’ |  |
| *B4galt6 R* | 5’-TGGCCTCTTTGAAACCC-3’ |  |
